# The diagnostic performance of International Ovarian Tumor Analysis: Simple Rules for diagnosing ovarian tumors—a systematic review and meta-analysis

**DOI:** 10.3389/fonc.2024.1474930

**Published:** 2025-01-20

**Authors:** Awadia Gareeballah, Moawia Gameraddin, Sultan Abdulwadoud Alshoabi, Amirah Alsaedi, Maisa Elzaki, Walaa Alsharif, Ibrahim Mohamed Daoud, Shrooq Aldahery, Magbool Alelyani, Elrashed AbdElrahim, Fahad H. Alhazmi, Zuhal Y. Hamd, Raga Ahmed Abouraida, Mayeen Uddin Khandaker, Mohamed Adam

**Affiliations:** ^1^ Department of Diagnostic Radiology, College of Applied Medical Sciences, Taibah University, Al-Madinah Al-Munawwarah, Saudi Arabia; ^2^ Faculty of Radiological Sciences and Medical Imaging, Alzaiem Alazhari University, Khartoum, Sudan; ^3^ Department of Obstetrics and Gynecology, Faculty of Medicine, Alneelain University, Khartoum, Sudan; ^4^ Department of Obstetrics and Gynecology, Batterjee Medical College (BMC), Abha, Saudi Arabia; ^5^ Department of Applied Radiologic Technology, College of Applied Medical Sciences, University of Jeddah, Jeddah, Saudi Arabia; ^6^ Department of Radiological Sciences, College of Applied Medical Sciences, King Khalid University, Abha, Saudi Arabia; ^7^ Radiological Sciences Department, College of Applied Medical Sciences, Taif University, Taif, Saudi Arabia; ^8^ Department of Radiological Sciences, College of Health and Rehabilitation Sciences, Princess Nourah bint Abdulrahman University, Riyadh, Saudi Arabia; ^9^ Centre for Applied Physics and Radiation Technologies, School of Engineering and Technology, Sunway University, Bandar Sunway, Malaysia; ^10^ Faculty of Graduate Studies, Daffodil International University, Savar, Bangladesh; ^11^ Department of Physics, College of Science, Korea University, Seoul, Republic of Korea

**Keywords:** adnexal mass, ultrasonography, International Ovarian Tumor Analysis Simple Rules, IOTA-SR, benign tumor, malignant tumor, gynecology

## Abstract

**Introduction:**

Adnexal masses are a common health issue in gynecology; the challenge lies in the differential diagnosis of these masses. The International Ovarian Tumor Analysis Simple Rules (IOTA-SR) offers the first scoring system to aid in diagnosis. It is based on a set of five ultrasound imaging features indicative of a malignant ovarian tumor and five features indicative of a benign tumor. This review aims to assess the diagnostic performance of IOTA-SR for classifying ovarian tumors as benign or malignant.

**Methods:**

A systematic review was conducted on MEDLINE, Embase, Google Scholar, Scopus, and Web of Science. The terminologies “IOTA-SR”, “adnexal, mass”, and “ovarian tumors scoring” were employed. Twenty-seven research articles conducted from 2008 to 2022 were included in the meta-analysis; the publication outcome indicates that performance quality tests were extracted directly or indirectly, including true positive (TP), false positive (FP), true negative (TN), and false negative (FN). The Quality Assessment of Diagnostic Accuracy Studies 2 (QUADAS-2) was used to evaluate the study quality and estimate the risk of bias. After estimating the pooled effect of the sensitivity, specificity, and diagnostic odds ratio (DOR), the summary receiver operating characteristic (SROC) curve was estimated using the bivariate random effects model. Utilizing Cochran’s Q statistics and Higgins’s inconsistency test through the I^2^ index for pooled analysis, the heterogeneity of studies was quantitatively evaluated. The funnel plot and Egger’s test were utilized to visually and quantitatively evaluate potential publication bias.

**Results:**

Among 27 studies, including 7,841 adnexal masses, the results of this meta-analysis showed excellent diagnostic performance with a pooled sensitivity of 92% [95% confidence interval (CI), 0.89–0.94] and a pooled specificity of 92% (95% CI, 0.89–0.94). The IOTA-SR was applicable in 85.7% of adnexal masses.

**Conclusion:**

The IOTA-SR is highly effective in the presurgical differentiation of malignant versus benign adnexal masses when applied by an expert ultrasonography operator.

## 1 Introduction

Ovarian masses are occasionally found incidentally, and distinguishing between benign and malignant ovarian masses is crucial for determining the best course of treatment. Ovarian cancer accounts for 3.7% of all cancers in women and is the seventh most prevalent type (1). Five to ten percent of women will have surgery for a suspected ovarian tumor at some point in their lives (2). The age-adjusted incidence rate in Europe in 2012 was 13.1 per 100,000 women, with a total of 65,538 newly reported cases. Because it can be fatal, ovarian cancer is a significant health issue in gynecology, accounting for 42,700 fatalities in Europe in 2012 (mortality rate, 7.6 per 100,000) (3).

Why is the early detection of ovarian cancer crucial? The majority of ovarian malignancies are epithelial ovarian cancer (EOC) that develops rapidly, so early diagnosis is urgent. Immediate referral to a gynecologic oncologist for a prompt diagnosis of the mass’s nature can ensure more effective treatment (4). Treatment strategy can be altered by determining preoperatively whether an adnexal mass is benign or malignant, but this is often the most challenging step because currently accessible diagnostic tests are not entirely reliable. Radiological imaging and tumor markers are the most frequently used procedures, but the need for improvement cannot be overstated.

Previous studies have reported that between 5% and 40% of adnexal cysts are malignant, meaning that other patients whose cysts are classified as benign or ambiguous may wait longer for treatment rather than being referred for unneeded surgery (5, 6). The International Ovarian Tumor Analysis (IOTA) group suggested modifying the simple rules risk (SRR) technique to increase its predictive value (7). All kinds of adnexal masses can have the likelihood of their malignancy calculated using this logistic regression model.

Imaging methods, such as ultrasonography (USG), computed tomography (CT), and magnetic resonance imaging (MRI) can be used to assess soft tissue structure, development, and lymph nodes. The first-line examination before surgery for ovarian masses is ultrasound—either abdominal or transvaginal—the most commonly used tool for detecting pelvic and abdominal pathology. This non-invasive imaging technique can accurately distinguish between normal and malignant adnexal masses (8). Therefore, several recommendations and recognized reports relying on sonographic characterization have been proposed to determine the likelihood of ovarian masses being malignant. The IOTA group’s Simple Rules (IOTA-SR), developed in 2008, is one such recommendation widely used in clinical practice (9). The IOTA-SR includes five ultrasonography characteristics that can differentiate benign from malignant tumors. Benign tumor traits (B-features) include B1 = unilocular, B2 = presence of solid portions with the greatest solid component < 7mm in diameter, B3 = presence of sonic shadows, B4 = smooth multilocular tumor with ≤ 10 cm its largest diameter, and B5 = no blood supply (color score 1). Malignant tumor traits (M-features) include M1 = irregular solid tumor, M2 = presence of ascites, M3 = presence of four or more papillary structures, M4 = irregular multilocular tumor with ≥10 cm maximum diameter, and M5 = very strong blood flow (color score 4) (1).

Subsequently, a CT scan and an MRI are performed to check for nodal involvement, the breadth of the disease in the upper abdominal region, the structure of the masses, and whether there is any uncertainty regarding the origin of the mass, such as the gastrointestinal tract, urinary system, or retroperitoneum. Subjective pattern recognition assessment using USG is operator-dependent because it relies on the operator’s experience. Furthermore, there needs to be more in the USG-based explanation of the adnexal masses’ terminology and classification standards. Different classification systems have been developed to overcome these limitations that use USG findings and other modalities to distinguish adnexal masses. These prompted the development of various scoring systems for differentiating benign from malignant adnexal tumors, including the IOTA-SR (10, 11).

Several studies conducted in the literature mentioned that the smaller sample size limit the generalizability of diagnostic performance of IOTA-SR in diagnosing ovarian tumors, by pooling the data from several studies, meta-analysis affords a more robust estimate of sensitivity, specificity and diagnostic accuracy.

The authors note that few studies have been conducted in the Gulf and low-income countries, pointing to a gap in knowledge and practice using IOTA-SR in the diagnosis and differentiation of ovarian masses. Filling this gap in knowledge and practice is vital. This study was conducted to evaluate the efficacy of IOTA strategies for diagnosing ovarian masses using a larger sample size. In the view of the authors, it was effective, simple, and easy to apply in clinical practice with excellent performance.

## 2 Methodology

### 2.1 Sources of information and search strategy

**Figure 1:**
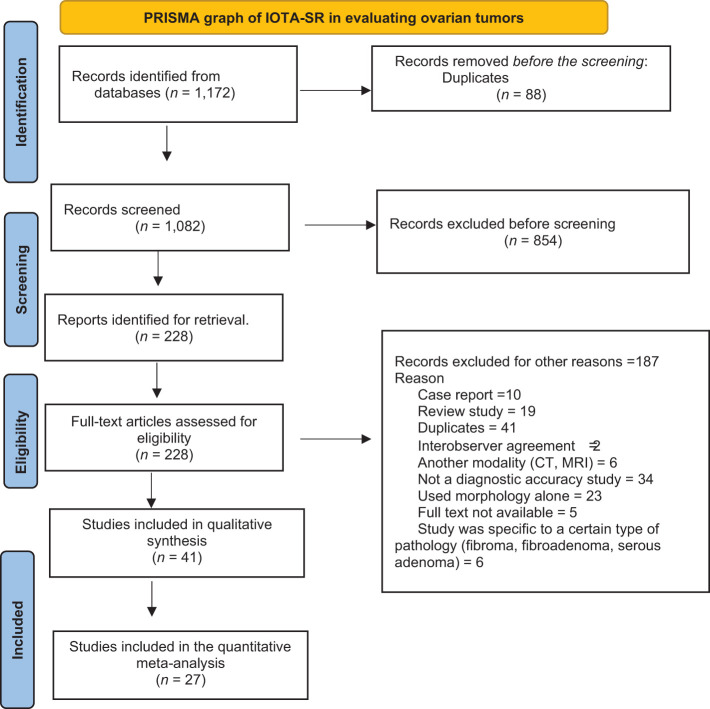
Flowchart of selection of studies for the meta-analysis to assess the performance of IOTA-SR in discriminating malignant ovarian tumors. IOTA-SR, International Ovarian Tumor Analysis Simple Rules.

A systematic search was performed in relevant databases, including PubMed, Scopus, Google Scholar, Web of Science, Embase, and MEDLINE, for articles that evaluated the accuracy of IOTA-SR in diagnosing ovarian masses. The following terms were searched: “IOTA”, “adnexal tumor”, and “ovarian cancer scoring”. The initial search strategy’s results were filtered using a title and abstract screening process. The titles of 1,172 studies were extracted. Only 228 were identified for further screening. Of those, 187 were excluded for various reasons, and 27 were included in the meta-analysis. **Figure 1** illustrates the results of our systematic search and meta-analysis.

### 2.2 Eligibility criteria

Two authors read and examined all relevant articles to determine those meeting the inclusion and exclusion criteria for this meta-analysis. The inclusion criteria were 1) women with ovarian tumors; 2) ovarian cancer was diagnosed via IOTA and histopathology; 3) outcome indicated performance quality of testing, including specificity and sensitivity; and 4) rates of true positive (TP), false positive (FP), false negative (FN), and true negative (TN) could be inferred from the study. The exclusion criteria were case reports, systematic reviews, lack of original data, duplicate data, and conflicting outcome indicators.

### 2.3 Data extraction

From the selected studies, the extracted data were entered separately by the same two authors. These consisted of the first author’s name, publication year, study design (prospective vs. retrospective), blind method (blind from the reference standard vs. unreported), number of patients, number of malignant tumors, number of benign tumors, sensitivity, specificity, TP, FP, FN, and TN, which were gathered and tabulated.

### 2.4 Quality assessment

#### 2.4.1 Assessment of risk bias

The included studies’ risk of bias and applicability were evaluated by two authors (AG and AA) using the Quality Assessment of Diagnostic Accuracy Studies 2 (QUADAS-2) instrument (12). This instrument uses a series of questions across four domains, each of which can be answered yes, no, or unclear, to assess the whole range of bias present in the included studies’ design and execution. The following domains are represented in QUADAS-2 items. 1) Patient selection: This domain assesses the risk to diagnostic accuracy of bias in the selection of participants for the study, including the methods used to recruit and enroll participants and the criteria used to include or exclude them. It included the following questions: a) Was the patient sample enrolled in sequential or random order? b) Is a case–control study avoided? c) Has the study been prevented from unnecessary exclusion? d) Were the patients chosen typical of the participant group to which the test index would apply?

2) Index test: This domain assesses the risk of bias in how the index test (the diagnostic test being evaluated) was performed, interpreted, and reported. It consisted of the following question: Were the outcome of the index test results evaluated without identifying the results of the gold standard?

3) Reference standard: This domain assesses the risk of bias in how the reference standard (the gold standard used to diagnose the condition of interest) was performed, interpreted, and reported and included two questions: a) Is the reference standard a good indicator to classify the target status correctly? b) Were the standard reference findings interpreted without identifying the findings of the index test?

4) Flow and timing: This domain assesses the risk of bias in the flow of patients during the study and the duration of the test index and gold standard relative to each other and included four questions: a) Was there a suitable interval between index test(s) and the gold standard? b) Were all patients provided a reference standard? c) Did all patients have access to the same gold standard? d) Were all participants included in the analysis?

**Figure 2:**
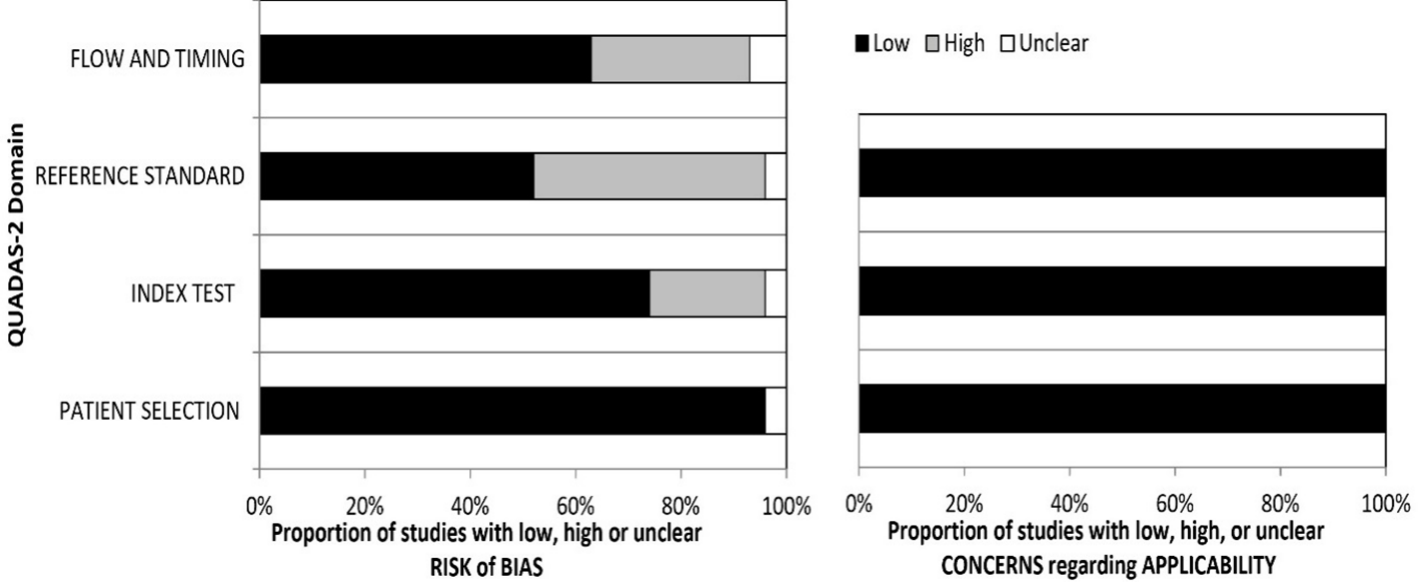
Summary graph of the QUADAS-2 assessment in included studies. QUADAS-2, Quality Assessment of Diagnostic Accuracy Studies 2.

The application assessment is conducted using three domains: a) the individuals’ selection domains (Is there a concern that the included patients do not match the review question)?, b) the index test domain (Does it matter if the index test, its conduct, or its interpretation differs from the review question)?, and c) the gold standard (Is there a concern that the target condition, as defined by the standard of reference, does not match the review question)? (see **Figure 2**).

### 2.5 Statistical analysis

The diagnostic performance of IOTA-SR in classifying benign or malignant ovarian tumors was evaluated. The analysis strategy used was similar to that described by Shim et al., in which the bivariate random effects model was used to estimate the pooled effect of sensitivity, specificity, and diagnostic odds ratio (DOR), followed by an estimation of the summary receiver operating characteristic (SROC) curve. The heterogeneity of the studies was quantitatively evaluated using Cochran’s Q statistics and Higgins’s inconsistency test through the I^2^ index for pooled analysis. If Cochran’s Q statistics p-value is <0.05 and the I^2^ is >50%, these are considered evidence of substantial heterogeneity. Possible publication bias was evaluated quantitatively and visually using the funnel plot and Egger’s test, respectively, with a p-value of <0.10 indicating significant asymmetry and the presence of publication bias. This analysis was conducted by employing the “Mada” and “meta” packages in the R statistical software (Version 1.4.1103, R Foundation for Statistical Computing, Vienna, Austria). Meta-regression was used to explain the heterogeneity in the results. The DOR was used to assess publication bias and heterogeneity, as it depends significantly on sensitivity and specificity.

## 3 Results

The study was conducted on 7,841 ovarian masses included in the publications reviewed, approximately 72.2% of which were benign and 27.8% were malignant on histopathology (**Table 1**).

**Table 1:** Type of ovarian tumors according to histopathology results.

Type	Number	%
Benign	5,664	72.2
Malignant	2,177	27.8
Total cases	7,841	100

The total number of studies included was 27. Only one study was conducted on pregnant women (32). Four studies were included twice in the analysis, resulting in 31 total studies. Dakhly et al. perform two step analysis, one step with inconclusive masses were excluded and another step with inconclusive included and considered as malignant. de Gauna et al. analyzed two centers, A and B. Knafel et al. analyzed level 1 and 2 IOTA-SR studies based on examiner experience, and Hidalgo et al. used the simple rule descriptor in Steps 1 and 2) (21, 23, 26, 36).

**Figure 3:**
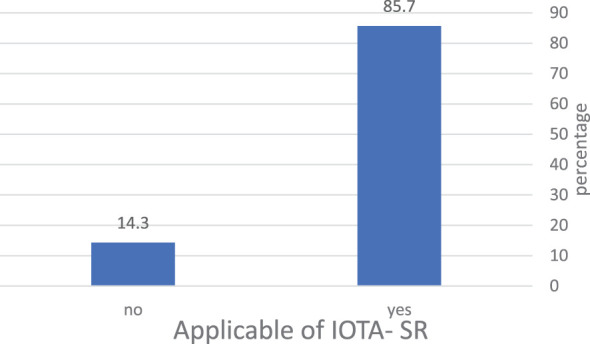
The percentage of masses included in the application of IOTA-SR in the diagnosis of ovarian tumors. IOTA-SR, International Ovarian Tumor Analysis Simple Rules.

IOTA-SR was applicable in 85.7% of the total cases included in the studies. The total number of malignant tumors was 1,971, and 4,889 were benign, based on histopathology. With regard to IOTA-SR, the number of TPs was 1,795, TNs 4,457, FPs 432, and FNs 176, as shown in **Table 2** and **Figure 3**.

**Table 2:** Frequency of total and number of cases in which IOTA-SR was applied and the performance of IOTA-SR in the diagnosis of malignant tumors.

No.	Author	Applicable of IOTA	Total cases	TP	FN	FP	TN
1	Timmerman 2008 (9)	386	507	106	6	25	249
2	Timmerman 2010 (13)	1,501	1,938	340	29	49	1,083
3	Fathallah 2011 (14)	109	122	8	3	3	95
4	Hartman 2012 (15)	91	103	20	2	9	60
5	Alcalzar 2013 (16)	270	340	29	4	6	231
6	Sayasneh 2013 (17)	214	255	46	7	3	158
7	Feharsal 2016 (18)	119	119	57	1	16	45
8	Nunes 2014 (19)	237	303	101	4	15	117
9	Tantipalakorn 2014 (20)	319	389	88	19	10	202
10a	Knafel 2014 (SR1) (21)^***^	186	226	58	3	7	118
10b	Knafel 2014 (SR2) (21)^***^	206	226	64	4	9	129
11	Tinnangwattana 2015 (22)	94	100	25	3	11	55
12a	de Gauna 2015 (A) (23)^++^	114	135	27	0	4	62
12b	de Gauna 2015 (B) (23)^++^	109	133	11	2	4	92
13	Garg 2017 (24)	45	50	11	1	5	28
14	Auekitrungrueng 2019 (25)	479	392	98	19	22	253
15a	Dakhly 2019 (inconclusive malignant) (26)^***^	396	396	88	6	83	219
15b	Dakhly 2019 (26)^***^	292	396	44	6	22	220
16	Shetty 2019 (27)	183	205	26	11	2	144
17	Solanki 2020 (10)	174	174	30	1	11	133
18	Vilà Famada 2020 (28)	74	102	16	1	4	47
19	Sharma 2020 (29)	57	61	13	1	3	40
20	Sibangi 2020 (30)	70	80	23	1	2	44
21	Mohammad 2021 (31)	60	60	36	2	3	19
22	Czekierdowski 2021 (32)^**^	36	36	9	0	17	10
23	Phinyo 2021 (33)	392	479	98	19	22	253
24	Bamniya 2021 (34)	124	124	24	2	6	92
25	Mongan 2021 (35)	29	29	18	2	4	5
26a	Hidalgo 2021 (SR) (36)^***^	54	100	11	1	1	41
26b	Hidalgo 2021 (SR three-step strategy) (36)^***^	100	100	16	1	2	81
27	Xie 2022 (37)	453	453	254	15	52	132

IOTA-SR, International Ovarian Tumor Analysis Simple Rules; TP, true positive; FN, false negative; FP, false positive; TN, true negative.

^**^Conducted in pregnant women.

^***^Two-step analysis.

^++^Conducted in two centers.

### 3.1 Assessment of QUADAS-2

The summary graph of the QUADAS-2 assessment for the selected publications is shown in **Figure 2**. The four areas (domains) depicted on the bias risk graph are the patient selection domain, the index test domain, the reference, and the domain of flow and timing. The concerns regarding applicability in the graph are represented in three domains: the gold standard, the index test, and patient selection. In both the risk of bias and the applicability of data, patient selection showed low concerns. However, in some studies, there are concerns regarding the reference standard and index test, as some classified borderline tumors as malignant in IOTA classifications, while one study considered borderline malignant in histopathology.

### 3.2 Results of diagnostic performance of IOTA- SR in diagnosing ovarian tumors

**Figure 4:**
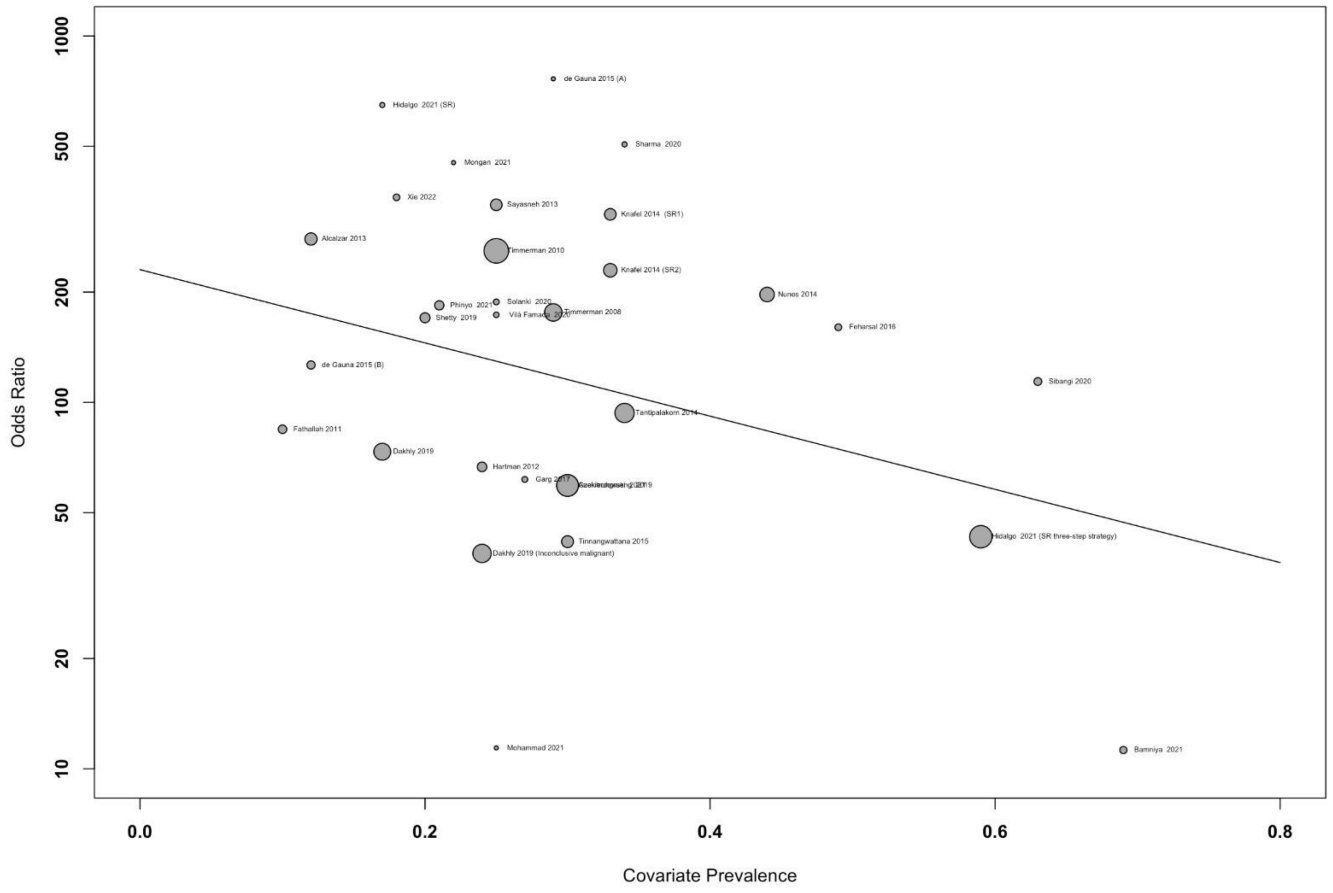
Meta-regression graph showing the diagnostic odds ratio (DOR) against accuracy of the study, with the size of bubble representing the study weight.

In the meta-regression graph, **Figure 4** shows the bubble plot, DOR on the y-axis, and prevalence on the x-axis. The bubble’s size represents the study weight. The gradient of the line was −0.02 [95% confidence interval (CI), −0.001 to −0.05], suggesting that the DOR decreased insignificantly by 0.02 per one unit of prevalence rate (p = 0.05).

**Figure 5:**
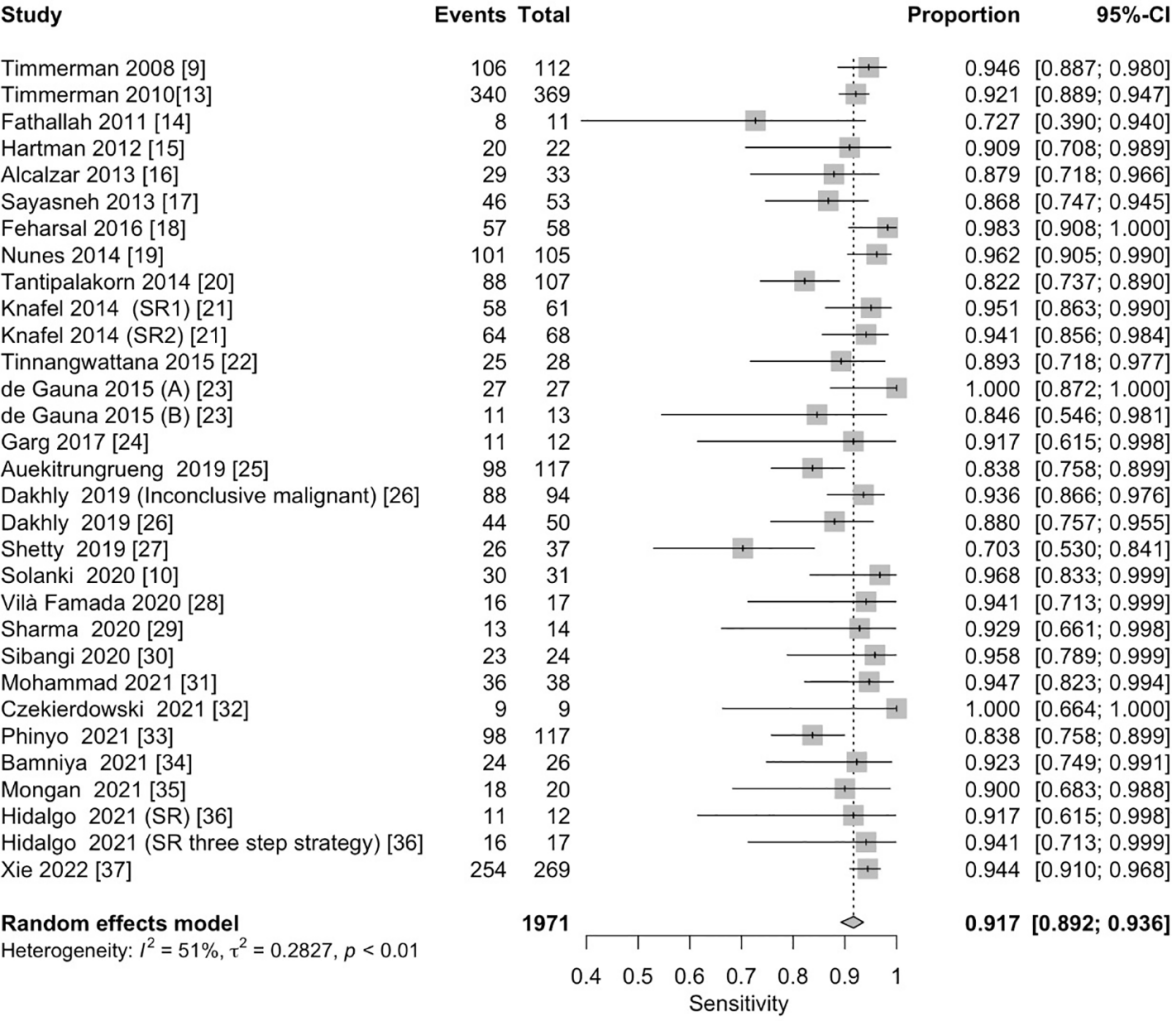
Forest plot of the pooled sensitivity of the IOTA Simple Rules in the diagnosis of ovarian cancer. The dotted vertical line represents the pooled effect size point where the effect size in individual studies has a very different distribution (heterogeneity) around this line. The diamond at the bottom represents the pooled effect and its 95% CI. CI, confidence interval; IOTA, International Ovarian Tumor Analysis.

**Figure 6:**
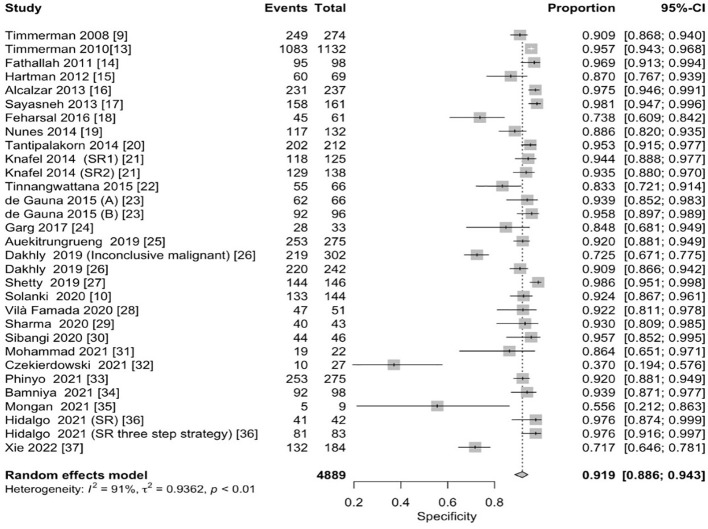
Forest plot of the pooled specificity of IOTA-SR in the diagnosis of ovarian cancer. The dotted vertical line represents the pooled effect size point where the effect size in individual studies has a very different distribution (heterogeneity) around this line. The diamond at the bottom represents the pooled effect and its 95% CI. CI, confidence interval; IOTA-SR, International Ovarian Tumor Analysis Simple Rules.

**Figure 7:**
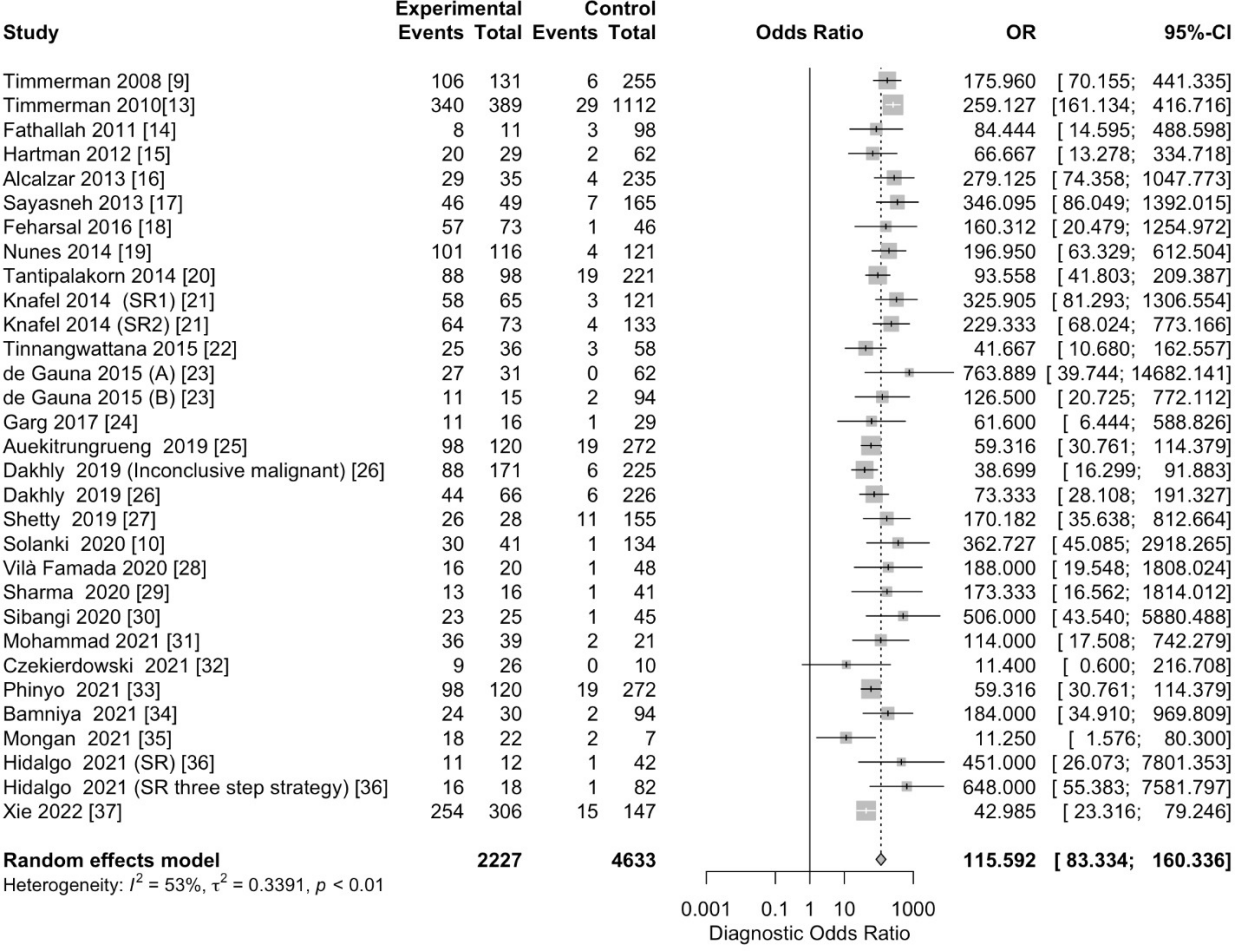
Forest plot of the pooled DOR of IOTA-SR in the diagnosis of ovarian cancer. The dotted vertical line represents the pooled effect size point where the effect size in individual studies has a very different distribution (heterogeneity) around this line. The diamond at the bottom represents the pooled effect and its 95% CI. OR, odds ratio; CI, confidence interval; DOR, diagnostic odds ratio; IOTA-SR, International Ovarian Tumor Analysis Simple Rules.

**Figure 8:**
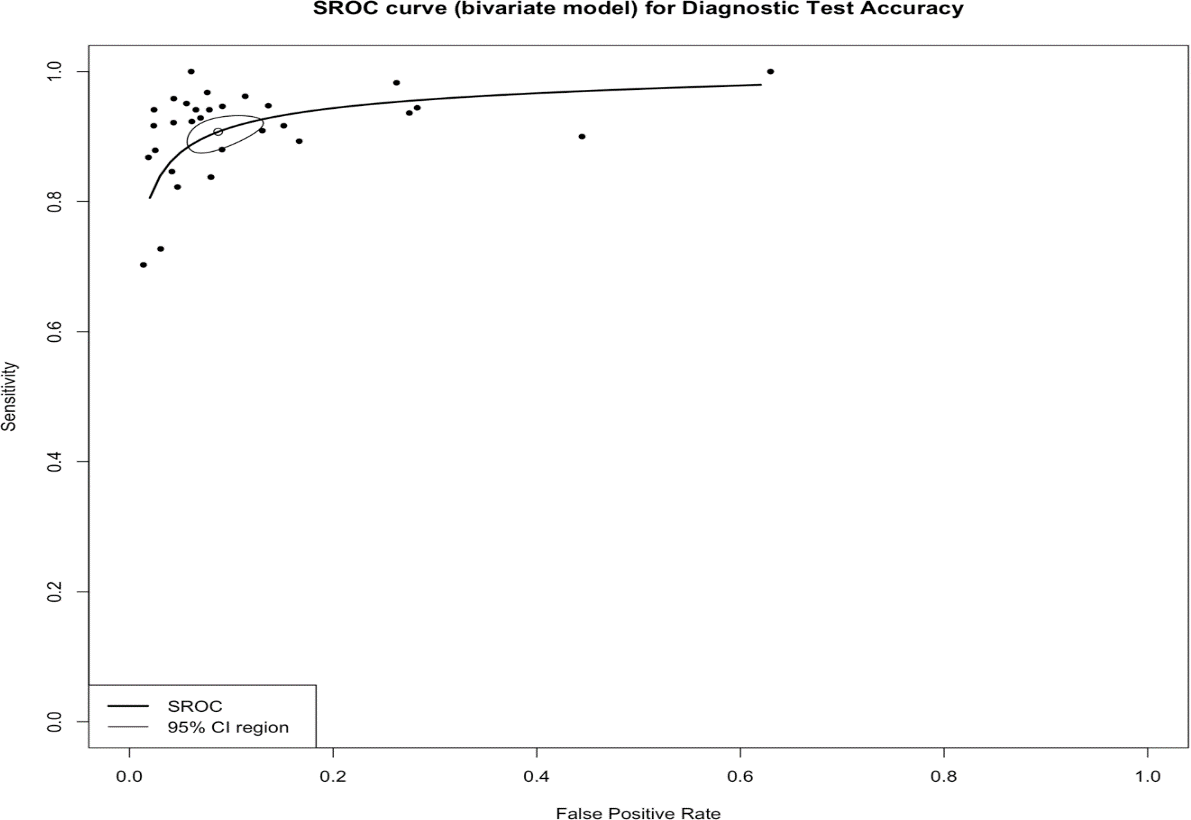
The summary receiver operating characteristic (SROC) curve of IOTA-SR in the diagnosis of ovarian cancer. CI, confidence interval; AUC, area under the curve; IOTA-SR, International Ovarian Tumor Analysis Simple Rules.

**Figure 9:**
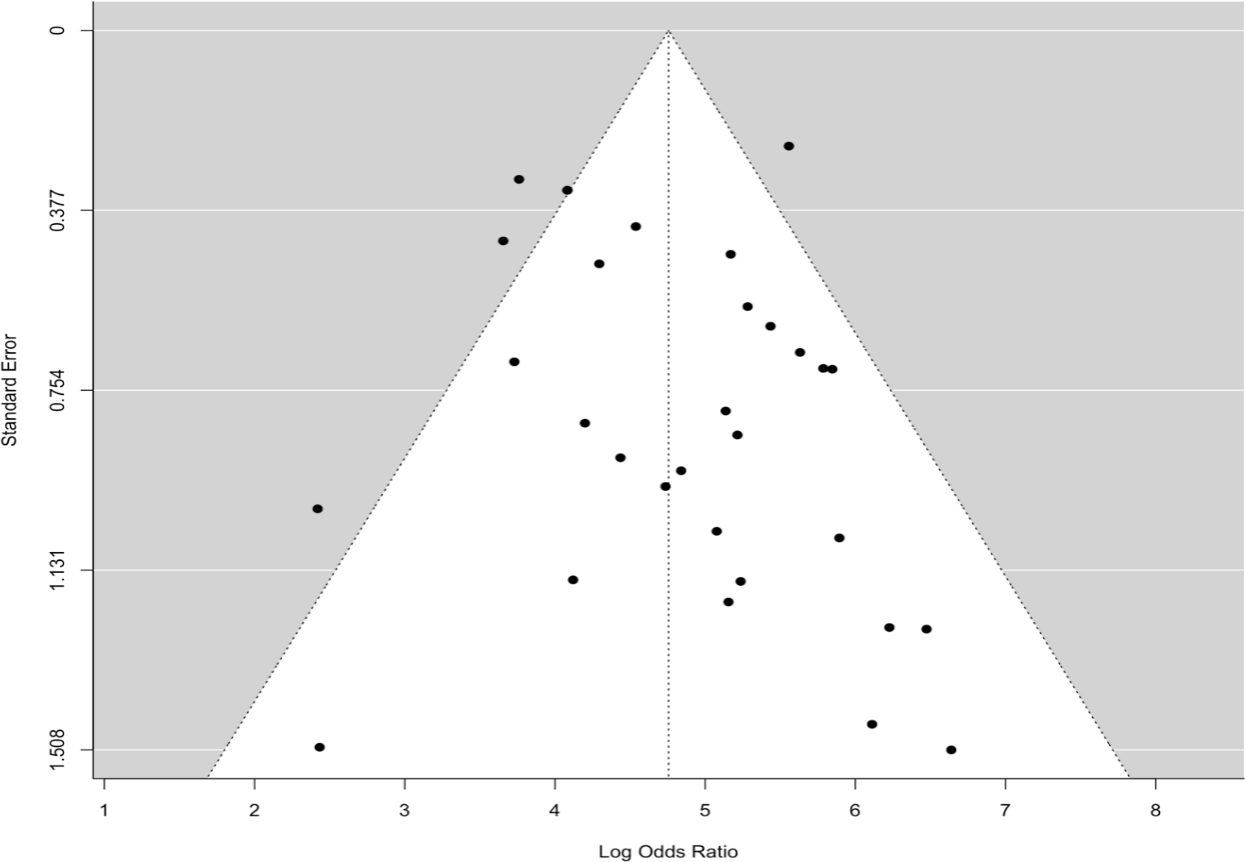
The funnel plot is symmetrical, showing the absence of publication bias.

The pooled sensitivity for the diagnosis of ovarian malignancies was 0.92 (95% CI, 0.89–0.94), as shown in **Figure 5**, while the pooled specificity was 0.92 (95% CI, 0.89–0.94) (**Figures 6**, **7**). The DOR was 115.59 (95% CI, 83.33–160.34), as shown in **Figure 8**. **Figure 8** displays a ROC curve with an area under the curve (AUC) of 0.95 (I^2^ = 5.3% in the abstract). In the sensitivity, specificity, and DOR analyses, the I^2^ was 50.7%, 90.6%, and 52.9%, respectively, while Cochran’s Q statistics p value was <0.01 in all, indicating heterogeneity. The generated funnel plot in **Figure 9** was symmetrical (Egger’s test, p = 0.19), indicating the absence of publication biases.

## 4 Discussion

The differential diagnosis of adnexal masses is a recognized problem in gynecological practice. To this end, statements on imaging, biomarkers, and prediction models for the preoperative diagnosis of ovarian cancers were collaboratively developed by the European Society of Gynaecological Oncology, the International Society of Ultrasound in Obstetrics and Gynecology, the IOTA group, and the European Society for Gynaecological Endoscopy (38). In this study, the efficacy of IOTA Simple Rules in differentiating benign from malignant adnexal tumors was investigated. The accuracy of IOTA in evaluating ovarian tumors was found to be relatively high.

Many USG technologies for the diagnosis of adnexal masses have been introduced worldwide. The introduction of imagistic exploration has hastened and enhanced USG diagnoses. The gynecologist must apply the IOTA to evaluate benign, borderline, and malignant adnexal masses (39, 40). The IOTA study is the most extensive work on the accuracy of USG in diagnosing ovarian tumors. Ovarian tumors have been studied to better understand their morphology and duplex characteristics. The IOTA group was founded 15 years ago to develop a reliable algorithm for diagnosing ovarian cancer. The IOTA-SR is the first scoring system to rely on a set of five USG features indicative of a malignant ovarian tumor (M-features) and five USG features indicative of a benign tumor (B-features) (1).

In this review, the diagnostic performance of IOTA-SR was assessed for classifying benign or malignant ovarian tumors. It was found that the pooled sensitivity was 92% (95% CI, 0.89–0.94) and that the pooled specificity was 92% (95% CI, 0.89–0.94). These results align with a previous meta-analysis by Nunes et al., who reported that the IOTA-SR is a reliable triage test for USG operators with varying experience levels and has a pooled sensitivity of 93% and a specificity of 95% when they are applicable (19). This finding is compatible with a previous meta-analysis with a large sample size of adnexal masses that reported 93% and 80% sensitivity and specificity for IOTA-SR, respectively (41). Another previous study on the external validation of IOTA-SR reported 94.3% and 94.9% overall sensitivity and specificity, respectively (42). A previous study reported that subjective assessment based on USG and IOTA may offer superior value to tumor markers, such as cancer antigen 125 (CA-125) and human epididymis protein 4 (HE4) assessment in complex adnexal masses (43). Furthermore, another study reported that the IOTA-SR had a high diagnostic accuracy compared to the risk of malignancy index (RMI) in distinguishing benign from malignant ovarian tumors (25).

Despite the excellent performance and simplicity of IOTA-SR, they have a significant limitation: they cannot be applied in all cases of adnexal masses (11). Tantipalakorn et al. reported that the IOTA-SR could be applicable in 80.1% of adnexal masses (20), and Lee et al. reported that IOTA-SR could be applicable in 80% of adnexal masses but was inconclusive (1). Nunes et al. reported that IOTA-SR was applicable in 78.2% with 96.2% sensitivity and 88.6% specificity (19). These meta-analysis results showed that IOTA-SR was applicable in 85.7% of adnexal masses in the included studies.

Ultimately, for accurate presurgical diagnosis of adnexal masses, the American College of Obstetricians and Gynecologists recommends “a multivariable approach by combining demographic, clinical, imaging, and laboratory parameters to perform the best diagnosis” (44).

Several studies have assessed the performance of IOTA-SR in combination with biomarkers to enhance the diagnostic accuracy of ovarian mass assessments. A study on 479 pre- and postmenopausal women reported that a combination of CA-125 and IOTA-SR models had better diagnostic value in differentiating malignant from benign ovarian tumors with AUCs of 0.94 and 0.98, respectively (33). Jha et al. found that the utilization of both IOTA Simple Rules and CA-125 exhibited exceptional diagnostic efficacy in distinguishing between benign and malignant ovarian tumors, surpassing the use of either CA-125 or IOTA Simple Rules individually (AUC of 0.94 for the combination tool; IOTA-SR + CA-125) (45). A study conducted by Xie et al. to assess the efficacy of the IOTA Simple Rules, Ovarian-Adnexal Reporting and Data System (O-RADS), and CA-125 in discerning between benign and malignant adnexal masses was examined. The findings indicated no notable variance in diagnostic precision when employing a combination of two approaches, specifically, IOTA-SR and CA-125 versus O-RADS and CA-125. As a result, the authors suggest using either IOTA-SR or O-RADS in conjunction with CA-125 to distinguish between benign and malignant lesions prior to surgery (37). Other studies have found that the integration of IOTA Simple Rules with established tumor markers (CA-125 and HE4) enhances the precision of diagnosing malignant ovarian masses before surgery (46).

The main strength of the current study is that it includes many previous studies without significant bias, as indicated in the analysis (**Figure 9**), thus improving diagnostic accuracy. However, the study must acknowledge a few limitations because heterogeneity was found among the studies selected, as evidenced by Cochran’s Q statistics; inconsistencies and disparities in the study population’s characteristics and variations in the percentage of malignancies across the selected population were the main contributors to this heterogeneity.

There is a lack of studies in the Gulf and low-income countries on the application of IOTA-SR in ovarian tumors, which underscores a gap in knowledge and practices on this topic. The IOTA-SR criteria significantly influence international literature; these criteria offer good sensitivity and specificity in distinguishing between benign and malignant ovarian tumors. They reduce the need for expensive diagnostic procedures, such as MRI or CT, guide patient care, and empower medical professionals to opt for surgery, monitoring, or further imaging, ultimately improving patient outcomes. The education and training of ultrasound practitioners on IOTA-SR reduce interobserver variability and encourage uniform diagnostic procedures across regions. The IOTA’s clinical significance is supported by this systematic review and meta-analysis, serving as a roadmap for future research in ovarian cancer, particularly in the Gulf and low-resource countries.

## 5 Conclusion

In conclusion, this meta-analysis demonstrated that IOTA-SR is highly effective in the presurgical differentiation of malignant from benign adnexal masses. The IOTA Simple Rules is an effective test for detecting ovarian malignancies with a pooled sensitivity of 92% (95% CI, 0.89–0.94) and a pooled specificity of 92% (95% CI, 0.89–0.94).
